# Image-Based Artificial Intelligence for Predicting Malignant Transformation of Oral Potentially Malignant Disorders: A Scoping Review

**DOI:** 10.3390/jcm15145623

**Published:** 2026-07-17

**Authors:** Shaul Hameed Kolarkodi, Faraj Alotaiby, Mohammed Fakhry Almutairy, Syed Fareed Mohsin, Safia Shoeb Shaikh, Minal Vaibhav Awinashe, Mohamed Abdulcader Riyaz, Suresh Kandagal Veerabhadrappa

**Affiliations:** 1 Department of Oral and Maxillofacial Diagnostic Sciences, College of Dentistry, Qassim University, Buraydah 52571, Saudi Arabia; f.alotaiby@qu.edu.sa (F.A.); m.almutairy@qu.edu.sa (M.F.A.); s.syedabdulmohsin@qu.edu.sa (S.F.M.); sa.shaikh@qu.edu.sa (S.S.S.); m.awinashe@qu.edu.sa (M.V.A.); m.salahudeen@qu.edu.sa (M.A.R.); 2 Department of Oral Diagnostic Sciences, Faculty of Dentistry, SEGi University, Petaling Jaya 47810, Malaysia; dr.suri88@gmail.com

**Keywords:** scoping review, PRISMA-ScR, radiomics, pathomics, deep learning, convolutional neural network, transformer, oral potentially malignant disorders, oral leukoplakia, oral epithelial dysplasia

## Abstract

**Background:** Oral potentially malignant disorders (OPMDs) have a complex, but not consistently consistent, risk of transformation to oral squamous cell carcinoma (OSCC). The histopathologic grading of dysplasia is the traditional means of prognosis although there is considerable inter-observer variability and the tool has poor predictive value. Thus, image-based AI-based methods such as computational pathomics (CP), clinical-photograph deep learning (CDL), and optical or spectroscopic image analysis via machine learning (ML) or deep learning (DL) have been proposed as potential non-invasive risk stratification methods. Aim of the review is to identify the current state of the evidence for AI applications in the field of image-based diagnosis and treatment of OPMDs, their methodological characteristics and predictive accuracy, and priorities for future research. **Methods:** the scoping review was conducted using Joanna Briggs Institute methodology and PRISMA-ScR guidelines. The PubMed/MEDLINE, Scopus, Web of Science and Embase databases were searched between January 2018 and March 2026. Inclusion criteria were studies that used quantitative image analysis, ML or DL to diagnose, prognosticate, or stratify risk of OPMD in OPMD cohorts. A pre-piloted form was used to extract data which were then synthesized descriptively. **Results:** the 423 records identified included 24 (16 primary image-based studies and 8 contextual reviews) that met the inclusion criteria. The majority of studies were retrospective (15/16), and computational pathomics (*n* = 10), clinical-photograph deep learning and optical/spectroscopic imaging (*n* = 4) were emphasized. There was no study that used the conventional radiologic radiomics (CT, MRI, CBCT, or PET/CT) in OPMD cohorts. Overall, predictive performance was good, with AUROC values ranging from 0.73 to 0.96 for the detection of malignant transformation, 0.94 to 0.97 for OPMD versus OSCC discrimination, and 0.90 to 0.97 for dysplasia grading. Only 44% contained external validation, only 6% were prospective, and only limited adherence was made to IBSI, TRIPOD-AI and CLAIM standards. **Conclusions:** AI-based image recognition for OPMDs shows good predictive performance, and it has yet to be developed to a mature stage of the process. Future multicentre study, image standardisation, external validation and better reporting standards are needed. Remarkably, radiologic radiomics is an important and unexplored research gap in OPMD risk prediction.

## 1. Introduction

Oral potentially malignant disorders (OPMDs) are a heterogeneous collection of lesions of the oral mucosa that have an increased, although not uniform, risk of developing into oral squamous cell carcinoma (OSCC). Under this term are grouped the following conditions: oral leukoplakia, oral leukoplakia of HPV origin, oral submucous fibrosis (OSMF), erythroplakia (EP), proliferative verrucous leukoplakia (PVL), oral lichen planus (OLP), oral lichenoid lesions (OLL), oral graft-versus-host disease (oGvHD), palatal lesions in reverse smokers, actinic cheilitis, and oral dysplastic lesions of HPV origin [1]. The pooled estimates of MT for oral leukoplakia have been approximately 1–3% per year, and there have been reports of increased MT for proliferative verrucous leukoplakia and erythroplakia. A significant proportion of the global burden of oral cancer is borne by LMICs, as they are the region where OPMDs are common and typically not screened. Population screening and clinical surveillance of OPMDs can reduce OSCC mortality; the impact of this is small [2]. The inter- and intra-observer variance (κ values of 0.22–0.66) of the prognostic tool used in routine practice and the fact that it only moderately predicts MT in the individual patient make histopathological grading of dysplasia a less reliable prognosis. This has led to a great deal of research focusing on objective, measurable, and non-invasive markers that might help to further refine individual risk stratification and determine the level of intervention.

A solution that has been proposed is radiomics. The original definition of radiomics is about the extraction of quantitative features from radiological images (CT, MRI, PET, CBCT) and the application of these features in clinical decision-support models [3,4]. In this review it is broader operational definition quantitative or learned image features from any modality used to image oral tissue. We do this because we do not want to have any confusion about what is meant by “image features”: some are qualitative and some are quantitative. In the head-and-neck field, traditional radiomics has been applied to investigate nasopharyngeal carcinoma [5], characterize the extension of tongue cancer on MRI [6], and create prognostic biomarkers for head-and-neck SCC [7]. The term “radiomics” has expanded to referring to quantitative extraction of features from any digital image of biological tissue, ranging from whole slide haematoxylin and eosin (H&E) images (known as pathomics) to engineered features from clinical photographs to deep-learning features from optical or spectroscopic images (e.g., optical coherence tomography (OCT) and Fourier-transform infrared (FTIR) spectroscopy). We have been forced to use this more general definition because it is the definition found in the existing literature on OPMD methodology, and because it allows for the review to cover the entire literature on OPMD methodology and not just the radiological subset.

While some of the work mentioned previously [8,9] has been published before, the current evidence map has become considerably larger since 2022, with imaging-based AI applied to OPMDs in a multitude of ways and modalities. In this context, the question is broad, the goal is methodological characterisation and gap identification, and the different types of imaging modalities, modelling techniques and outcomes make it unsuitable to pool effect sizes.

### Review Questions

This scoping review addresses four primary questions:

Q1. Which imaging modalities have been used in radiomics- and AI-based studies of OPMDs, and how is the operational definition of “radiomics” applied across studies?
Q2. What feature-extraction approaches and ML/DL algorithms (including support vector machines (SVM), random forests (RF), artificial neural networks (ANN), convolutional neural networks (CNN), transformer-based architectures and graph-based models) have been applied?
Q3. What predictive performance has been reported for the prediction of malignant transformation, dysplasia grade and OPMD-versus-OSCC classification?
Q4. What is the state of external validation, reporting standardisation (IBSI v2.0, TRIPOD-AI, CLAIM) and methodological quality across the included literature, and what gaps remain?

## 2. Materials and Methods

### 2.1. Protocol and Registration

This scoping review was carried out according to the Joanna Briggs Institute (JBI) methodology [10], which is an adaptation of the [11] framework as modified by [12]. The reporting is based on the Preferred Reporting Items for Systematic Reviews and Meta-Analyses Ex-tension for Scoping Reviews (PRISMA-ScR) checklist [13] and is summarized on the checklist in Appendix B. The protocol was developed beforehand and modified following a pilot calibration using 20 records. That pilot led to three changes: (i) the list of eligible imaging modalities was broadened to take in optical and spectroscopic modalities (OCT, confocal laser endomicroscopy, FTIR, Raman-spectroscopy) and clinical photographs; (ii) charting fields were added to record adherence to the IBSI and TRIPOD-AI reporting standards; and (iii) the requirement for OPMD-specific outcomes in mixed OPMD/OSCC cohorts was clarified. There were no other changes to eligibility. The complete protocol is given in Supplementary Materials.

### 2.2. Eligibility Criteria

Eligibility was designed following the Population—Concept—Context framework recommended by [10] for scoping reviews.

Population. Patients with oral potentially malignant disorders (OPMDs) (Oral leukoplakia, erythroplakia, proliferative verrucous leukoplakia, oral submucous fibrosis, oral lichenoid lesions, oral lichen planus, oral epithelial dysplasia and actinic cheilitis) as defined by the WHO consensus nomenclature [1]. Cohorts with mixed outcome reports of both OSCC and OPMD were included only if OPMD-specific outcomes were also provided. Studies without comparator group or without OPMD follow up were excluded. The larynx, pharynx and oesophagus were not separately specified as anatomical sites.

Concept. Application of quantitative image analysis (radiomics, pathomics, or any deep-learning pipeline taking an image as input) to an OPMD population. Imaging modalities were computed tomography (CT), magnetic resonance imaging (MRI), CBCT, PET/CT, ultrasound, optical coherence tomography (OCT), in vivo confocal laser endomicroscopy (CLE), narrow-band or autofluorescence imaging, FTIR or Raman spectroscopy, and standard or smartphone clinical photographs. Engineered radiomic (classical ML like SVM, random forests, gradient-boosted trees, logistic regression, shallow ANNs) and end-to-end deep learning (CNNs, vision transformers, graph neural networks, multimodal architectures including images and EMR data) methods were eligible.

Context. Aims for OPMDs are either diagnostic or prognostic or risk stratification, and the primary outcome is either malignant transformation to OSCC or presence or grade of dysplasia or OPMD-versus-OSCC discrimination or OPMD-versus-normal-mucosa discrimination. Studies focused solely on treatment response and those focused on surgical planning/imaging data not associated with OPMDs were excluded.

Study designs. Systematic and scoping reviews published (charted evidence context) and primary studies (retrospective, prospective, cross-sectional, case control). Editorials and narrative commentaries, abstracts from conferences without full-text, animal-only studies, simulation-only methodology papers, and preprints were excluded. The published studies included in this review were conducted between 1 January 2018 and 31 March 2026, thus covering the modern era of radiomics and deep learning.

### 2.3. Information Sources

PubMed/MEDLINE (National Library of Medicine (NLM) interface), Scopus (Elsevier, Amsterdam, The Netherlands), Web of Science Core Collection (Clarivate, Philadelphia, PA, USA) and Embase (Amsterdam, The Netherlands) were searched electronically. We also conducted backward citation searching in the reference lists of all included reviews and four key primary studies cited by the included reviews [9,14,15,16] well as forward citation searching in Google Scholar for the four most cited prior reviews [9,14,15,16] and the seminal radiomics methodology paper [3]. The methodological focus of the review did not include searching for grey literature.

### 2.4. Search Strategy

The search strategy was built iteratively with an information specialist and validated against a set of 12 known-relevant studies before use. To reflect the expanded scope, the image-concept block combined generic AI/radiomics terms with modality-specific terms (clinical photographs, smartphone imaging, OCT, autofluorescence, narrow-band imaging, confocal laser endomicroscopy, FTIR, Raman, spectroscopy, whole-slide imaging and digital pathology). The full PubMed strategy, adapted for syntax in Scopus, Web of Science and Embase and reproduced in full in Appendix A, was as follows:

(“radiomics”[tiab] OR “radiomic”[tiab] OR “texture analysis”[tiab] OR “machine learning”[tiab] OR “deep learning”[tiab] OR “convolutional neural network*”[tiab] OR “artificial intelligence”[tiab] OR “computational pathology”[tiab] OR pathomics[tiab] OR “clinical photograph*”[tiab] OR smartphone[tiab] OR “optical coherence tomography”[tiab] OR OCT[tiab] OR autofluorescence[tiab] OR “narrow-band imaging”[tiab] OR “confocal laser endomicroscopy”[tiab] OR FTIR[tiab] OR Raman[tiab] OR spectroscopy[tiab] OR “whole-slide imaging”[tiab] OR “digital pathology”[tiab]) AND (“oral potentially malignant”[tiab] OR OPMD[tiab] OR leukoplakia[tiab] OR erythroplakia[tiab] OR “submucous fibrosis”[tiab] OR “lichen planus”[tiab] OR “oral lichenoid”[tiab] OR “oral epithelial dysplasia”[tiab] OR “actinic cheilitis”[tiab]) AND (“malignant transformation”[tiab] OR “oral cancer”[tiab] OR “oral squamous cell carcinoma”[tiab] OR carcinoma[tiab] OR dysplasia[tiab] OR prognos*[tiab] OR risk[tiab])

Date filter: 1 January 2018–31 March 2026. Language: English. Final searches were executed on 31 March 2026.

### 2.5. Study Selection Process

**Figure 1:**
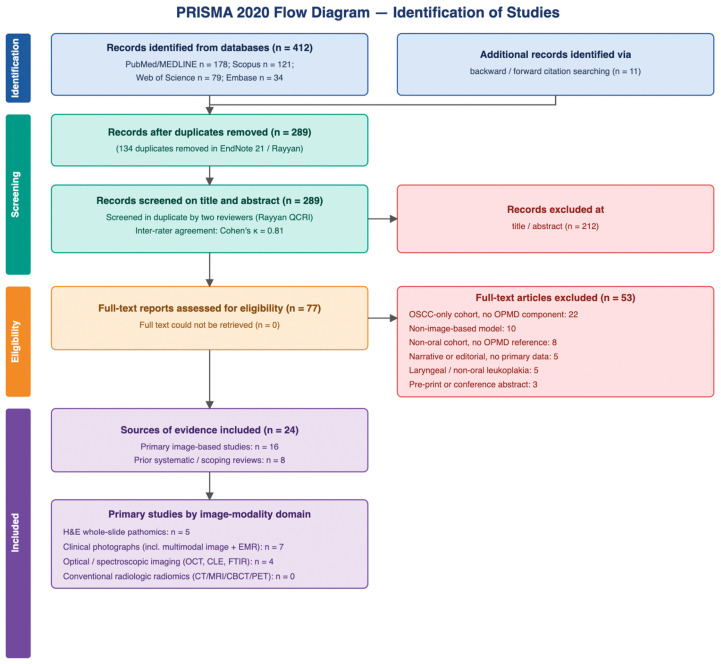
PRISMA-ScR flow diagram of the search and selection process. Sources of evidence cascade from 412 identified records through de-duplication, title-and-abstract screening, full-text retrieval and final inclusion. Adapted from [13].

Records were exported to RIS format, deduplicated in EndNote 21 (Philadelphia, PA, USA) and uploaded to Rayyan (Qatar Computing Research Institute, Doha, Qatar) for double screening. Titles and abstracts were independently screened by two reviewers. The records that seemed to meet the eligibility criteria were retrieved in full and compared to the detailed eligibility criteria. Full records of potentially eligible children were retrieved and checked against the full eligibility criteria. Any disagreements were resolved through discussion or, if not, by a third reviewer. Interrater reliability was substantial (Cohen’s κ = 0.81 (95% CI 0.73–0.89)) based on the first 100 records in the queue of each reviewer at the point of title/abstract screening. Table 1 and Figure 1 report search yield and the inclusion cascade, respectively, reported by database.

### 2.6. Data Charting

The first five eligible studies were independently charted by two reviewers, which led to a pre-piloted charting form based on the standard scoping-review charting form developed by [10] being refined. Bibliographic information (authors, year, country, journal), setting/de-sign (single centre vs. multi-centre, retrospective vs. prospective), OPMD type, cohort size, imaging modality and protocol, segmentation approach (manual, semi-automated, fully automated), feature-extraction pipeline (engineered radiomic features vs. learned representations), ML/DL algorithm class, primary outcome and reference standard, performance metrics reported (AUROC, sensitivity, specificity, accuracy, F1, C-index), internal validation strategy (hold-out, k-fold, nested CV), external validation status (same vs. different institution; geographical vs. temporal), follow-up duration for prognostic studies, adherence to standardisation initiatives (IBSI, TRI-POD-AI, CLAIM, STARD), availability of code or trained models, and reported limitations. Two reviewers independently charted each study and differences were resolved by consensus.

### 2.7. Critical Appraisal

**Figure 2:**
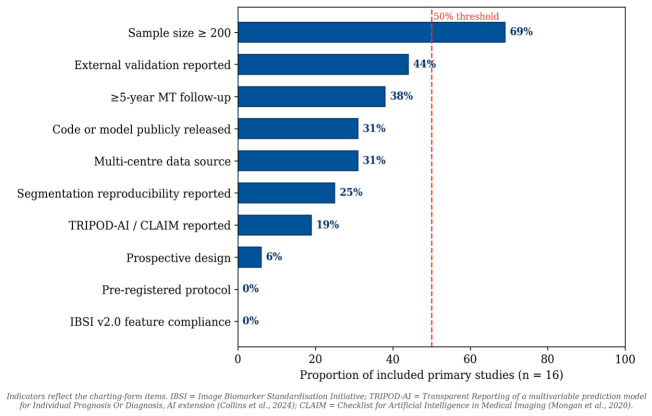
Methodological-quality indicators across the 16 included primary studies [6,18,19,20,21,22,23,24,25,26,27,28,29,30,31]. Bars show the proportion of studies that met each indicator, ordered from most to least frequently met. The dashed red line marks a 50% threshold for reference. Indicators reflect the charting-form items captured per Section 2.7. IBSI = Image Biomarker Standardisation Initiative; TRIPOD-AI = Transparent Reporting of a multivariable prediction model for Individual Prognosis Or Diagnosis, Artificial Intelligence extension; CLAIM = Checklist for Artificial Intelligence in Medical Imaging.

In line with JBI scoping-review methodology, we did not carry out a formal risk-of-bias appraisal of individual sources. Rather, a set of methodological indicators were documented in the charting form, including external validation, sample size, reproducibility of the segmentation and adherence to the TRI-POD-AI, CLAIM and IBSI reporting guidelines, in accordance with recent recommendations for scoping reviews for AI in medicine [17]. They jointly allow us to discuss, in an orderly fashion, the maturity of the evidence base. The methodological-indicator profile is summarised in Figure 2. A quantitative risk-of-bias scoring was not attempted.

### 2.8. Synthesis of Results

**Figure 3:**
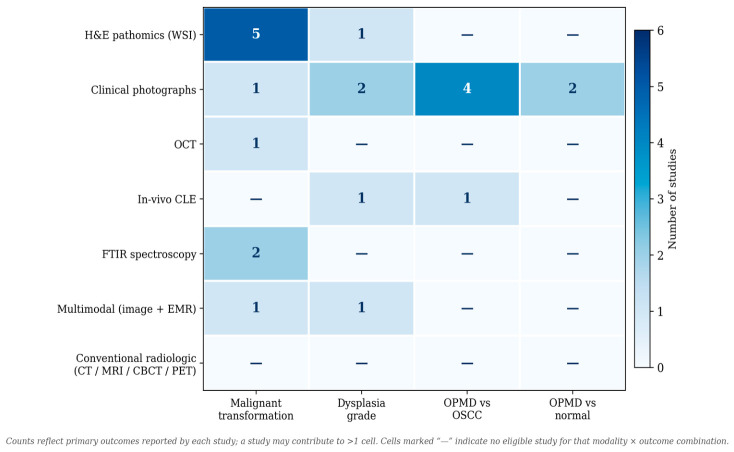
Evidence map showing the number of included primary studies (*n* = 16) [6,18,19,20,21,22,23,24,25,26,27,28,29,30,31] addressing each combination of imaging-modality domain (rows) and primary outcome (columns). Each study is counted once per modality–outcome combination it reports; a study addressing two outcomes therefore contributes to two cells, so cell values sum to more than the number of studies. Empty cells (shown as a dash) indicate that no eligible study was identified for that modality and outcome combination.

**Figure 4:**
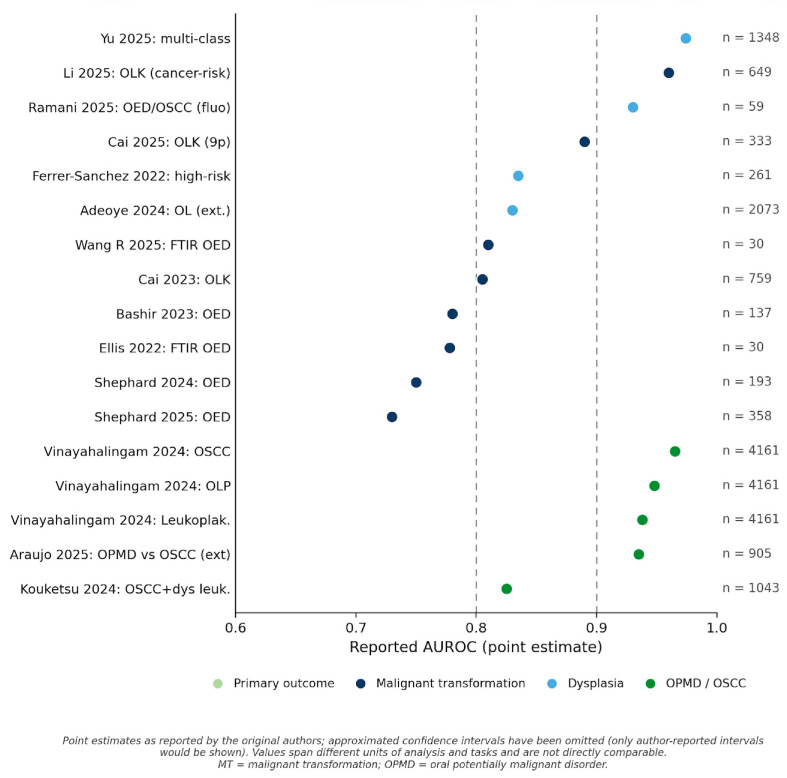
Performance landscape of the 16 included primary studies [6,18,19,20,21,22,23,24,25,26,27,28,29,30,31]. Each point represents the reported AUROC point estimate; approximated confidence intervals have been omitted, and only intervals explicitly reported by the original authors would be displayed. Because reported values span different units of analysis (image-, frame-, lesion- or patient-level) and different prediction tasks, they are not directly comparable. Studies are coloured by primary outcome category: malignant transformation (MT, dark blue), dysplasia grade (light blue), and OPMD-versus-OSCC discrimination (green). Dashed vertical lines mark the conventional 0.80 and 0.90 AUROC thresholds.

We carried out a descriptive, narrative, and synthesis based on the PRISMA-ScR recommendations [17]. The studies included were grouped in two ways: by imaging-modality domain (pathomics studies, clinical photography studies, optical/spectroscopic imaging studies, multimodal image-plus-EMR, and conventional cross-sectional radiologic imaging studies); and by primary outcome (malignant transformation, dysplasia grade, OPMD-vs-OSCC, and OPMD-vs-normal). Performance metrics are presented as reported by the original authors but do not follow scoping review methodology that would require a pooling of performance metrics. The visual synthesis consists of the PRISMA-ScR flow diagram (Figure 1), an evidence map of modalities against outcomes (Figure 2), a performance map of AUROC point estimates (Figure 3) (only author-reported intervals would be displayed as approximated intervals were not used), and a methodological-quality bar chart (Figure 4). The answers to these questions are placed on these maps in Section 3.

## 3. Results

### 3.1. Selection of Sources of Evidence

The database search returned 412 records (PubMed/MEDLINE 178, Scopus 121, Web of Science Core Collection 79, Embase 34), and backward and forward citation searching added a further 11, giving 423 records in total. After removing 134 duplicates (EndNote 21 and Rayyan), 289 unique records went forward to title-and-abstract screening; 212 were excluded as off-topic, leaving 77 for full-text assessment. Of these, 53 full texts were excluded, each assigned a single primary reason: OSCC-only cohort without an OPMD component (*n* = 22), non-image-based model; that is, clinico-pathological or molecular-only prediction models such as [32,33] (*n* = 10), non-oral cohort without an OPMD reference (*n* = 8), narrative or editorial without primary data (*n* = 5), laryngeal or non-oral leukoplakia (*n* = 5), and pre-print or conference abstract (*n* = 3). The final corpus comprised 16 primary image-based studies and 8 previous systematic or scoping reviews, giving 24 sources of evidence. Figure 1 shows the PRISMA-ScR flow and Table 2 lists the yields by database.

### 3.2. Characteristics of Included Sources of Evidence

Table 1 (in landscape orientation, after Section 3) provides the complete charting of the 16 studies included. Fifteen were retrospective and one was prospective [20] five were multi-centre and the rest single- or two-centre. The number of samples ranged from 30 [6] to 4161 photographs [29]. Geographically, the work is primarily from the United Kingdom, China (including Hong Kong), and Brazil; there is a single piece each from the United States, Australia, Spain, the Netherlands/Germany and Japan, and a single multi-country study: Hong Kong/India/Nigeria. The eight previous reviews included six systematic and two scoping reviews with none reproducing the combined PCC scope of the present review. The field is then widely dispersed, with no eligible primary study coming from the Eastern Mediterranean region, or from sub-Saharan Africa unless it was a collaborating site. That represents a significant geographical gap, especially in light of the regional epidemiology of OPMDs.

### 3.3. Findings by Review Question

#### 3.3.1. Q1: Imaging Modalities and Operational Definition of “Radiomics”

The studies that were included were grouped into the following four image-modality domains as summarised in Figure 2 (the evidence map). Whole-slide H&E image computational pathomics of OPMD biopsies was the most methodologically advanced area [21,25,27]. The largest number of studies was conducted in deep learning on standard clinical or smartphone photos of the oral mucosa [18,19,22,23,29] including one multimodal approach that combined clinical images with EMR features [19]. Optical and spectroscopic imaging (OCT, in vivo confocal laser endomicroscopy and FTIR spectroscopy) was clustered in a smaller group [6,20,26,34]. None of the eligible studies examined conventional cross-sectional radiologic imaging (MRI, CT, CBCT, or PET/CT) directly in an OPMD cohort with the outcomes of malignant transformation, dysplasia, or OPMD vs. OSCC. Most of the photographic or pathomic materials were used in the reviews that were supposed to be about “radiomics in OPMDs” [15,16,35,36]. This strongly relates to the operational definition of radiomics in the OPMD field as defined in the current literature: The literature has, in fact, redefined radiomics to quantitative-image-feature extraction in the radiological, pathological and optical fields, to which we will return in Section 4.1.

#### 3.3.2. Q2: Feature-Extraction and Modelling Approaches

There were two analytical paradigms that emerged. In the first, engineered-feature radiomics, Haralick-style grey-level co-occurrence-matrix texture descriptors [37], first-order intensity statistics and shape descriptors are extracted from the delineated region of interest, and fed into a classical ML algorithm, which is mainly used in the spectroscopic and OCT studies [6,26]. Some of the classical ML models used in this paradigm were support vector machines (SVM), random forests (RF), gradient boosted trees (XGBoost), logistic regression and shallow artificial neural networks (ANN). Photographic and pathomics studies were dominated by the second, end-to-end deep learning, which involved the model learning its own image features. These specific architectures encompassed convolutional neural networks (CNNs) such as EfficientNet-B2 and Inception-V3, DenseNet-169, Mask R-CNN, Single-Shot MultiBox Detector, vision transformer networks (the Swin Transformer from [18,29]), and pipelines for object detection (YOLOv11 in [30]). To date, the best reported AUROC for cancer-risk prediction (0.96) came from one multimodal architecture that combined clinical images with features from electronic-medical-records (EMR) [19]. A similar clinical/EMR only survival model charted as evidence context as opposed to as an image-based primary study, ref. [23] used survival-aware deep learners (DeepSurv, DeepHit, Cox-Time) to predict time-to-event MT and also found adding clinico-demographic data aids performance (c-index 0.95 internal, 0.82 external), reinforcing the benefits of multimodal designs.

#### 3.3.3. Q3: Reported Predictive Performance

The predictive performance is summarised in Figure 3 and is presented in tabular form in Table 1. The area-under-the-ROC values reported for the primary endpoint of malignant transformation ranged from 0.73 to 0.96 and the median value was approximately 0.80. Histopathology-based MT models gave AUROC values of 0.73 ([28], three external cohorts), 0.75 [25], 0.78 [27], 0.81 in the test set and 0.89 for 9p-loss prediction as an MT biomarker. The value was the highest for the multimodal image-plus-EMR model ([19]: cancer-risk AUROC = 0.96), and the clinical/EMR-only survival model charted as context [23] had an internal c-index of 0.95 and external c-index of 0.82. Reported AUROC values generally had higher values (0.88–0.97), as the task of dysplasia presence or grade is comparatively easier [18], ext. AUROC = 0.83; [20], AUROC 0.90–0.96; [29], AUROC = 0.974; [22], AUROC = 0.948 for OLP and 0.938 for leukoplakia. The externally validated AUROC for discrimination between OPMD and OSCC on clinical photographs was 0.935 [30]. These numbers should be interpreted with caution, taking into account the patterns of validation outlined below.

#### 3.3.4. Q4: External Validation, Reporting Standards and Methodological Quality

The quality of the methodology and validity of the results is reported in accordance with external standards. Figure 4 provides an overview of methodological-quality indicators, combined into aggregates. Of the sixteen primary studies included, seven (44%) reported some level of external validation (evaluated on a cohort that was not used for the development and tuning, preferably at a different centre). Externally validated work is from the pathomics studies of the ODYN/OMTscore programme [27,28], the Hong Kong leukoplakia photograph and pathomics streams [19,21,23], and [30]. A small 56% (9/16) used internal cross validation only. Eleven of sixteen studies (69%) had a development cohort of ≥200 patients or images, and six studies (38%) had ≥5 years follow-up, which is the time needed for meaningful outcomes with regard to malignant-transformation. More crucially, none of these studies reported any compliance to the Image Biomarker Standardisation Initiative (IBSI v2.0) feature definitions in a format recommended for radiomics studies and only 3 of 16 (19%) referenced the TRIPOD-AI checklist [38] or the CLAIM checklist [39]; only 1 of 16 was prospective (6%) and none was pre-registered on a public protocol registry (0%). Only 25% of the articles reported on the reproducibility of the segmentation, and only 5 of 16 articles published their code or trained models publicly (31%). Overall, the image emerges of largely existing designs, lacking full external validation and an almost complete lack of pre-registered protocols and IBSI compliance. This combination is the biggest methodological problem with the current database.

## 4. Discussion

### 4.1. Summary of Evidence

The aim of this scoping review was to identify the extent of the current knowledge on image-based AI in the field of OPMDs in the last eight years. The majority of OPMD research relies on five computational pathomics studies that use H&E whole slide images, seven deep learning studies (including one multimodal paper comprising image and EMR), and a very small stream (*n* = 4) of work on optical or spectroscopic approaches. Conventional radiologic radiomics, the paradigm behind the original [3] formulation, is basically absent from the OPMD literature (*n* = 0). The diversity of imaging modalities and analytical pipelines demonstrates the versatility of OPMD-AI, extending beyond any one definition of radiomics. In practice, it is the application of any image-feature pipeline that has been done quantitatively or learned to the images of the oral mucosa. To avoid the conceptual ambiguity mentioned by the reviewers of the present work, we recommend that future reviews and primary studies explicitly make this operational definition. The evidence map (Figure 2) demonstrates that the densest cells are those of pathomics × malignant transformation (*n* = 5) and clinical-photographs × OPMD-vs-OSCC (*n* = 4); radiologic radiomics × any outcome (*n* = 0) and several non-photographic OPMD-vs-normal combinations remain empty.

### 4.2. Implications

There are three implications of the charted evidence. First, the most developed applications are dysplasia grading and distinguishing OPMD from OSCC, and the performance has been excellent (AUROC > 0.90 in several externally validated models such as [18,29,30], and the inputs are already widely used in clinical practice. Lack of discrimination does not imply clinical readiness. Given that the rate of malignant transformation is low, a model with a high AUROC may fail to detect high-risk lesions, and sensitivity, negative predictive value, calibration and operating threshold of the model should be considered in making a decision about deployment, not the AUROC. Potential areas for possible use involve triage of any suspicious oral lesion in primary or non-specialist care, guidance for biopsy, risk stratified surveillance of oral leukoplakia and proliferative verrucous leukoplakia and prioritisation of referral to oral medicine or head and neck oncology. Second, the most clinically useful application, namely true prediction of malignant transformation, is more challenging methodology-wise: long follow-up cohorts are required, and to date the AUROC for the models based on histopathology alone is 0.73–0.78, while the AUROC for the multimodal model can be as high as 0.96 with external validation. Third, cross-sectional radiologic radiomics is not just a research gap in OPMD cohorts. The majority of OPMDs will be mucosal lesions with no need for a cross-sectional imaging study, which is why it is not clinically indicated for early mucosal disease; this is partly due to appropriate clinical practice and the lack of benefit from cross sectional imaging. In selected cases, imaging is indicated (where there is a suspicion of invasion or deep extension, advanced oral submucous fibrosis, ambiguous clinical findings, or suspected early OSCC) and imaging will provide a clear opportunity for primary research on OPMD cohorts using CBCT, contrast enhanced ultrasound or non-ionising MRI radiomics, so long as the ethics of over-investigating a population with an annual MT rate of approximately 1–3% are respected. Retrospective radiomic extraction from existing image archives is a low-burden and ethically sound approach when radiologic imaging has already been clinically indicated (for example, when CBCT is required for advanced OSMF to assess the degree of bone involvement or staging MRI is indicated in the case of suspicious leukoplakia).

### 4.3. Limitations

Several caveats need to be kept in mind when reading this scoping review. First, the literature search might have failed to identify some regionally important work particularly from populations that are endemically affected by submucous fibrosis, mainly in South Asia, the Middle East and parts of South-East Asia where research is published in languages other than English, and not indexed in the databases searched. Second, scoping reviews are not meant to be used to get pooled effect estimates, so readers seeking a summary discriminative performance should refer to the available meta-analyses [14,15,19,39] which are all charted here as evidence context. Third, although guidance for conducting a formal critical appraisal of individual sources was not included in the JBI scoping-review guidance [10], key methodological indicators were captured during charting of the sources and are displayed in Figure 4. Fourth, the methods in this area, especially vision-transformer and foundation-model architectures, are progressing rapidly, and what is being reviewed at any given time is necessarily a snapshot. Fifth, the reported performance metrics cover a variety of tasks and units of analysis (image-, frame-, lesion- and patient-level) and are reported as point estimates only, and therefore absolute comparisons between studies should be made with care. Sixth, we have provided an empirically driven definition of “radiomics,” which extends to “pathomics” and “clinical-photograph deep learning.” This definition could be subject to reasonable doubt from other reviewers.

### 4.4. Future Research

Future primary research priorities include the following: (i) prospective multicentre cohorts with standardised photographic and/or cross-sectional imaging protocols, with at least 5 years of follow-up for malignant transformation, and closure of the gap observed in Figure 4 (15% prospective, multicentre, standardised photographic protocols; and 19% follow-up time > 5 years); (ii) explicit adherence to IBSI v2.0 for engineered radiomic features and to TRIPOD-AI and CLAIM for reporting prediction models, to close the methodological gaps seen in Figure 4 (15% explicit adherence to IBSI v2.0; 31% adherence to TRIPOD-AI and CLAIM); and (iii) routine geographical and demographic validation of all clinically promising models, before and after deployment, including LMIC settings, since image-only models have been shown to perform worse than multimodal ones [19,23] (to close the gap observed in Figure 4).

## 5. Conclusions

Evidence is still emerging and immature for image-based AI application in OPMDs, and the field is still developing. In an intentional departure from the traditional radiologic definition of “radiomics”, we included computational pathomics and clinical-photograph deep learning in addition to traditional radiological feature extraction, which may be different from the radiologic definition [3]; future OPMD studies should clarify which definition is being used. The reported predictive performances for malignant transformation range from AUROC 0.73–0.96, but they are based on the majority (69%) of retrospective single- or two-centre studies, with less than half (44%) of studies reported externally and very few studies (0%) that adhered to IBSI, TRIPOD-AI and CLAIM reporting standards. To inform routine OPMD management in a responsible way, studies that are standardised, prospective, validated by outside experts and transparently reported are required. This review provides a survey of the existing evidence and methodological quality landscape, including the most advanced applications (dysplasia grading, OPMD vs. OSCC discrimination from clinical photos and H&E images), which are not (yet) clinically validated for deployment, and a clearly defined research gap: cross-sectional radiologic radiomics in OPMD cohorts and cross-registered, multimodal, prospective MT-prediction studies.

## Figures and Tables

**Table 1:** Per-database search yields and the cascade of inclusion.

Database/Source	Records Identified	After De-Duplication	Passed Title/Abstract	Included in Synthesis
PubMed/MEDLINE	178	n/a	38	8
Scopus	121	n/a	18	6
Web of Science Core Coll.	79	n/a	6	3
Embase	34	n/a	4	2
Citation searching (manual)	11	n/a	11	5
Total identified	423	n/a	n/a	n/a
After EndNote/Rayyan de-dup	n/a	289	n/a	n/a
Records screened (title/abstract)	n/a	n/a	289	n/a
Full-text retrieved	n/a	n/a	n/a	77
Final included sources of evidence	n/a	n/a	n/a	24

“n/a” denotes that the operation does not apply to that row (de-duplication was performed across the merged corpus in EndNote 21 and confirmed in Rayyan QCRI; per-database de-duplication is therefore not separately reported). For the per-source rows (1–5), the columns “Passed title/abstract” (sum = 77) and “Included in synthesis” (sum = 24) attribute each record to the first source in which it was identified, so the column sums equal the cascade totals. Total records identified = 423 (412 databases + 11 citation searching).

**Table 2:** Characteristics of the 16 primary image-based studies included in the scoping review, charted by author/year, country, design, sample, OPMD population, imaging modality, feature-extraction and modelling approach, primary outcome and external validation [6,18,19,20,21,22,23,24,25,26,27,28,29,30,31,32,33]. Extended charting (unit of analysis, reference standard, number of malignant-transformation events, follow-up duration, validation type and whether image-derived features were used) is provided in Supplementary Table S1, and a per-study methodological-quality matrix in Supplementary Table S2. Reference numbers correspond to the reference list.

Author, Year (Country)	Design	Sample	OPMD/Population	Imaging Modality	Features & Model	Outcome & Key Metric	External Validation
Wang et al., 2025 (USA) [6]	Retrospective	30 OED biopsies; 180 spectra	OED	FTIR spectroscopy	ML classifier (benign vs. malignant risk)	Accuracy = 83.3%; F1 = 0.80	No
Yu et al., 2025 (China) [18]	Retrospective	1348 photographs	Normal vs. OL vs. OLP vs. OSMF vs. OSCC	Clinical photographs	Dual-stage Swin Transformer + DenseNet-169	5-class accuracy = 0.903, AUROC = 0.974	No
Li et al., 2025 (HK/China) [19]	Retrospective multicentre	598 train + 51 ext	Oral leukoplakia	Clinical images + EMR	OMMT-PredNet multimodal DL	AUROC = 0.92 (OED), 0.96 (cancer risk)	Yes
Ramani et al., 2025 (Australia) [20]	Prospective	59 patients/9168 frames	Dysplasia grades, OSCC	In vivo confocal laser endomicroscopy	Tandem Inception-V3 CNNs	AUROC 0.90–0.96	No
Cai et al., 2025 (China) [21]	Retrospective multicentre	333 OLK + 407 HNSCC	OLK & HNSCC	H&E WSI	Transformer + XGBoost (9p loss signature)	AUROC 0.890/0.825; MT prediction	Yes
Ferrer-Sánchez et al., 2022 (Spain) [22]	Retrospective single-centre	261 OL lesions	Oral leukoplakia	Digital photographs	U-Net + multi-task CNN	Sens 1.00, Spec 0.692	No
Adeoye et al., 2021 (HK/India/Nigeria) [23]	Retrospective multicentre	2073 photographs	Oral leukoplakia	Smartphone/clinical photos	EfficientNet-B2 CNN	AUROC = 0.882 (ext 0.828)	Yes
Cai et al., 2023 (China) [24]	Retrospective multicentre	759 H&E images	Oral leukoplakia	WSI histopathology	DL feature extraction + ML	AUROC 0.899 (val), 0.813 (test)	Yes
Bashir et al., 2023 (UK) [25]	Retrospective single-centre	137 OED	OED	H&E WSI	Weakly supervised DL	AUROC 0.78; C-index 0.73	No
Ellis et al., 2022 (UK) [26]	Retrospective	OED biopsies	OED	FTIR spectroscopy	Spectral ML model	Sens 79%, Spec 76%	No
Shephard et al., 2024 (UK) [27]	Retrospective two-centre	193 train + 89 ext	OED	H&E WSI	Nuclear segmentation + NN (OMTscore)	AUROC 0.75; recall 0.92	Yes
Shephard et al., 2025 (UK/Brazil) [28]	Retrospective multicentre	358 OED + 105 ctrl; 108 ext	OED	H&E WSI	Transformer ODYN pipeline	F1 = 0.96; AUROC = 0.73	Yes
Vinayahalingam et al., 2024 (NL/DE) [29]	Retrospective	4161 photographs	Healthy/OPMD/OSCC	Clinical photographs	Mask R-CNN + Swin Transformer	OSCC F1 = 0.852	No
Araújo et al., 2025 (Brazil) [30]	Retrospective two-centre	773 train + 132 ext	OPMD vs. OSCC	Clinical photographs	YOLOv11 + MobileNetV2	AUROC = 0.935 (external)	Yes
Kouketsu et al., 2024 (Japan) [31]	Retrospective single-centre	1043 images	Dysplastic OL & OSCC	Clinical photographs	SSD detector	Sens 83.7%, Spec 81.2%	No

Abbreviations: AUROC, area under the receiver-operating-characteristic curve; CLE, confocal laser endomicroscopy; CNN, convolutional neural network; EMR, electronic medical record; FTIR, Fourier-transform infrared spectroscopy; H&E, haematoxylin and eosin; HK, Hong Kong; HNSCC, head-and-neck squamous cell carcinoma; ML, machine learning; MT, malignant transformation; OED, oral epithelial dysplasia; OL, oral leukoplakia; OLK, oral leukoplakia; OLP, oral lichen planus; OPMD, oral potentially malignant disorder; OSCC, oral squamous cell carcinoma; OSMF, oral submucous fibrosis; sens, sensitivity; spec, specificity; WSI, whole-slide image.

## Data Availability

All data extracted from included studies are presented in Table 1 of this manuscript. The review protocol, the database-specific search strategies (Appendix A), the data-charting form, the extended charting and methodological-quality matrices (Supplementary Tables S1 and S2) and the completed PRISMA-ScR checklist (Appendix B) are provided as Supplementary Materials.

## Supplementary Materials

The following supporting information can be downloaded at: https://www.mdpi.com/article/10.3390/jcm15145623/s1, Table S1: Extended charting of the 16 primary image-based studies. Table S2. Per-study methodological-quality and risk-of-bias indicator matrix (*n* = 16).

## Appendix A. Database-Specific Search Strategies

Each database search was performed using the syntax appropriate to that database’s interface. The PubMed strategy is the canonical reference; the other strategies translate it as faithfully as possible while preserving the Boolean structure (radiomics/AI terms) AND (OPMD terms) AND (outcome terms).

### Appendix A.1. PubMed/MEDLINE (NLM Interface, 31 March 2026)

(“radiomics”[tiab] OR “radiomic”[tiab] OR “texture analysis”[tiab] OR “machine learning”[tiab] OR “deep learning”[tiab] OR “convolutional neural network*”[tiab] OR “artificial intelligence”[tiab] OR “computational pathology”[tiab] OR pathomics[tiab] OR “clinical photograph*”[tiab] OR smartphone[tiab] OR “optical coherence tomography”[tiab] OR OCT[tiab] OR autofluorescence[tiab] OR “narrow-band imaging”[tiab] OR “confocal laser endomicroscopy”[tiab] OR FTIR[tiab] OR Raman[tiab] OR spectroscopy[tiab] OR “whole-slide imaging”[tiab] OR “digital pathology”[tiab]) AND (“oral potentially malignant”[tiab] OR OPMD[tiab] OR leukoplakia[tiab] OR erythroplakia[tiab] OR “submucous fibrosis”[tiab] OR “lichen planus”[tiab] OR “oral lichenoid”[tiab] OR “oral epithelial dysplasia”[tiab] OR “actinic cheilitis”[tiab]) AND (“malignant transformation”[tiab] OR “oral cancer”[tiab] OR “oral squamous cell carcinoma”[tiab] OR carcinoma[tiab] OR dysplasia[tiab] OR prognos*[tiab] OR risk[tiab]) AND (“1 January 2018”[PDAT]: “31 March 2026”[PDAT]) AND English[lang]

### Appendix A.2. Scopus (Elsevier, 31 March 2026)

TITLE-ABS-KEY((radiomics OR radiomic OR “texture analysis” OR “machine learning” OR “deep learning” OR “convolutional neural network*” OR “artificial intelligence” OR “computational pathology” OR pathomics OR “clinical photograph*” OR smartphone OR “optical coherence tomography” OR OCT OR autofluorescence OR “narrow-band imaging” OR “confocal laser endomicroscopy” OR FTIR OR Raman OR spectroscopy OR “whole-slide imaging” OR “digital pathology”) AND (“oral potentially malignant” OR OPMD OR leukoplakia OR erythroplakia OR “submucous fibrosis” OR “lichen planus” OR “oral lichenoid” OR “oral epithelial dysplasia” OR “actinic cheilitis”) AND (“malignant transformation” OR “oral cancer” OR “oral squamous cell carcinoma” OR carcinoma OR dysplasia OR prognos* OR risk)) AND PUBYEAR > 2017 AND PUBYEAR < 2027 AND LANGUAGE(english)

### Appendix A.3. Web of Science Core Collection (Clarivate, 31 March 2026)

TS = ((radiomics OR radiomic OR “texture analysis” OR “machine learning” OR “deep learning” OR “convolutional neural network*” OR “artificial intelligence” OR “computational pathology” OR pathomics OR “clinical photograph*” OR smartphone OR “optical coherence tomography” OR OCT OR autofluorescence OR “narrow-band imaging” OR “confocal laser endomicroscopy” OR FTIR OR Raman OR spectroscopy OR “whole-slide imaging” OR “digital pathology”) AND (“oral potentially malignant” OR OPMD OR leukoplakia OR erythroplakia OR “submucous fibrosis” OR “lichen planus” OR “oral lichenoid” OR “oral epithelial dysplasia” OR “actinic cheilitis”) AND (“malignant transformation” OR “oral cancer” OR “oral squamous cell carcinoma” OR carcinoma OR dysplasia OR prognos* OR risk)); refined by Publication Years 2018–2026 and Languages = English

### Appendix A.4. Embase (Elsevier, 31 March 2026)

(‘radiomics’:ab,ti OR ‘radiomic’:ab,ti OR ‘texture analysis’:ab,ti OR ‘machine learning’:ab,ti OR ‘deep learning’:ab,ti OR ‘convolutional neural network*’:ab,ti OR ‘artificial intelligence’:ab,ti OR ‘computational pathology’:ab,ti OR ‘pathomics’:ab,ti OR ‘clinical photograph*’:ab,ti OR ‘smartphone’:ab,ti OR ‘optical coherence tomography’:ab,ti OR ‘oct’:ab,ti OR ‘autofluorescence’:ab,ti OR ‘narrow-band imaging’:ab,ti OR ‘confocal laser endomicroscopy’:ab,ti OR ‘ftir’:ab,ti OR ‘raman’:ab,ti OR ‘spectroscopy’:ab,ti OR ‘whole-slide imaging’:ab,ti OR ‘digital pathology’:ab,ti) AND (‘oral potentially malignant’:ab,ti OR ‘opmd’:ab,ti OR ‘leukoplakia’:ab,ti OR ‘erythroplakia’:ab,ti OR ‘submucous fibrosis’:ab,ti OR ‘lichen planus’:ab,ti OR ‘oral lichenoid’:ab,ti OR ‘oral epithelial dysplasia’:ab,ti OR ‘actinic cheilitis’:ab,ti) AND (‘malignant transformation’:ab,ti OR ‘oral cancer’:ab,ti OR ‘oral squamous cell carcinoma’:ab,ti OR ‘carcinoma’:ab,ti OR ‘dysplasia’:ab,ti OR ‘prognos*’:ab,ti OR ‘risk’:ab,ti) AND [2018–2026]/py AND [english]/lim

## Appendix B. PRISMA-ScR Checklist Mapping

The completed PRISMA-ScR checklist below maps each of the 22 reporting items of the PRISMA-ScR extension [13] to the section of this manuscript in which it is addressed. Table A1 presents the PRISMA-ScR checklist, indicating where each reporting item is addressed within the manuscript to demonstrate adherence to the reporting guidelines.

**Table A1:** PRISMA-ScR checklist mapping each reporting item to where it is addressed in this review.

Item	PRISMA-ScR Checklist Item	Reported in Section
1	Title: identifies as a scoping review	Title page
2	Structured summary	Abstract
3	Rationale for the review	Section 1 Introduction
4	Objectives and review questions	Section “Review Questions”
5	Protocol and registration	Section 2.1 Protocol and registration
6	Eligibility criteria (PCC)	Section 2.2 Eligibility criteria
7	Information sources	Section 2.3 Information sources
8	Search strategy	Section 2.4 Search strategy; Appendix A
9	Selection of sources of evidence	Section 2.5 Selection; Figure 1
10	Data charting process	Section 2.6 Data charting
11	Data items	Section 2.6 Data charting; Table 1
12	Critical appraisal of individual sources	Section 2.7 Critical appraisal
13	Synthesis of results	Section 2.8 Synthesis of results
14	Selection of sources of evidence (results)	Section 3.1 Selection; Figure 1
15	Characteristics of sources	Section 3.2 Characteristics; Table 1
16	Critical appraisal within sources	Section 3.3.4; Figure 4
17	Results of individual sources	Section 3.3.3; Table 1; Figure 3
18	Synthesis of results	Section 3.3; Figure 2, Figure 3 and Figure 4
19	Summary of evidence	Section 4.1 Summary of evidence
20	Limitations	Section 4.3 Limitations
21	Conclusions	Section 5 Conclusions
22	Funding	Declarations
