# Supplementary material for: Image-Based Artificial Intelligence for Predicting Malignant Transformation of Oral Potentially Malignant Disorders: A Scoping Review
Source: J Clin Med. 2026 Jul 17;15(14):5623. doi: 10.3390/jcm15145623 (PMC13412258; doi:10.3390/jcm15145623)
Supplement: Supplementary file 1 [file jcm-15-05623-s001.zip › jcm-4392996-supplementary.pdf]

Supplementary Table S1. Extended charting of the 16 primary image-based studies

Reference standard, unit of analysis, malignant-transformation (MT) events, follow-up, validation type and image-input status for each included primary study. NR = not reported in the source / not extractable from the primary report.

Study (year)	Unit of analysis	Reference standard	MT events / follow-up	Validation type	Image-derived input?
Shephard 2025	Slide / patient	OED grade + follow-up MT (histology)	NR / multi-cohort	External (3 centres)	Yes
Shephard 2024	Slide / patient	Follow-up MT (histology)	NR	External (2 centres)	Yes
Bashir 2023	Slide / patient	Follow-up MT (histology)	50 MT / NR	Internal 5-fold CV	Yes
Cai 2023	Image / patient	Follow-up MT (histology)	NR	External (separate test set)	Yes
Cai 2025	Slide / patient	9p chromosomal loss (molecular surrogate)	NR	External (multi-cohort)	Yes
Adeoye 2024	Image / patient	Histopathology (dysplasia)	NR	External (geographical)	Yes
Ferrer-Sánchez 2022	Lesion / patient	Follow-up MT + dysplasia grade	mean 5.5-y follow-up	Internal	Yes
Yu 2025	Image	Clinical / histology class label	NR	Internal	Yes
Vinayahalingam 2024	Image	Class label (histology-confirmed)	NR	Internal 5-fold CV	Yes
Araújo 2025	Image	Clinical / histology (OPMD vs OSCC)	NR	External (UFPB set)	Yes
Kouketsu 2024	Image / patient	Histology (OSCC / dysplastic)	NR	Internal	Yes
Yuan 2024	Image (OCT B-scan)	Prognostic outcome	NR	Internal (single centre)	Yes
Ramani 2025	Frame	Histology (dysplasia grade)	59 patients / 9,168 frames (prospective)	Internal	Yes
Ellis 2022	Spectrum / biopsy	Follow-up MT (histology)	NR	Internal	Yes
Wang R 2025	Spectrum / biopsy	Histology (OSCC vs benign)	30 biopsies / 180 spectra	Internal (nested CV)	Yes
Li 2025	Image + EMR / patient	Histology (OED) + cancer-risk follow-up	NR	External (51-case)	Yes (multimodal)

Supplementary Table S2. Per-study methodological-quality and risk-of-bias indicator matrix (n = 16)

Indicators supporting the aggregate proportions in Figure 4 and the methodological-risk summary requested by reviewers. ✓ = indicator met; ✗ = not met; NR = not reported.

Study	Ext. val.	Prosp.	Pt-level split	Data-leak. safeguard	Calibr.	Explain.	n ≥ 200	≥5-y FU	Multi-centre	Seg. reprod.	Code/model	TRIPOD/CLAIM	IBSI
Shephard 2025	✓	✗	✓	✓	NR	✓	✓	✓	✓	✓	✓	✓	✗
Shephard 2024	✓	✗	✓	✓	NR	✓	✓	✓	✗	✓	✓	✗	✗
Bashir 2023	✗	✗	✓	✓	NR	✓	✗	✓	✗	✓	✓	✗	✗
Cai 2023	✓	✗	✓	✓	NR	✗	✓	✓	✓	✗	✓	✗	✗
Cai 2025	✓	✗	✓	✓	NR	✓	✓	✗	✓	✗	✗	✗	✗
Adeoye 2024	✓	✗	✓	✓	NR	✗	✓	✗	✓	✗	✗	✓	✗
Ferrer-Sánchez 2022	✗	✗	✓	✓	NR	✗	✓	✓	✗	✓	✗	✗	✗
Yu 2025	✗	✗	✗	✗	NR	✗	✓	✗	✗	✗	✗	✗	✗
Vinayahalingam 2024	✗	✗	✗	✗	NR	✗	✓	✗	✗	✗	✗	✗	✗
Araújo 2025	✓	✗	✓	✓	NR	✗	✓	✗	✗	✗	✓	✗	✗
Kouketsu 2024	✗	✗	✓	✓	NR	✗	✓	✗	✗	✗	✗	✗	✗
Yuan 2024	✗	✗	✗	✗	NR	✗	✗	✗	✗	✗	✗	✗	✗
Ramani 2025	✗	✓	✗	✗	NR	✗	✗	✗	✗	✗	✗	✗	✗
Ellis 2022	✗	✗	✓	✓	NR	✓	✗	✓	✗	✗	✗	✗	✗
Wang R 2025	✗	✗	✓	✓	NR	✗	✗	✗	✗	✗	✗	✗	✗
Li 2025	✓	✗	✓	✓	NR	✗	✓	✗	✓	✗	✗	✓	✗

Column totals (✓ of 16): Ext. val. 7 (44%); Prosp. 1 (6%); Pt-level split 12 (75%); Data-leak. safeguard 12 (75%); Calibr. 0 (0%); Explain. 5 (31%); n ≥ 200 11 (69%); ≥5-y FU 6 (38%); Multi-centre 5 (31%); Seg. reprod. 4 (25%); Code/model 5 (31%); TRIPOD/CLAIM 3 (19%); IBSI 0 (0%).

Note: External validation, prospective design, sample size (≥200), ≥5-year follow-up and multi-centre status are coded directly from Table 1 and reproduce the proportions in Figure 4. The remaining indicators — patient-level split, data-leakage safeguard, calibration, explainability, segmentation reproducibility, code/model release and TRIPOD-AI/CLAIM — require the authors' original full-text charting sheet; the entries here are a reconstruction (calibration was not reported in any study; data-leakage safeguard is inferred from patient-level data splitting) and should be reconciled against the source coding before publication. ✓ = met; ✗ = not met; NR = not reported.
